# Supplementary material for: The rainfall effect onto solidification and stabilization of heavy metal-polluted sediments
Source: R Soc Open Sci. 2020 Jul 8;7(7):192234. doi: 10.1098/rsos.192234 (PMC7428253; doi:10.1098/rsos.192234)
Supplement: Support Information [file rsos192234supp1.docx]

**The rainfall effect onto solidification and stabilization of heavy metal- polluted sediments**

Yan Sun ^a^, Daofang Zhang ^a^*, Feipeng Li ^a^*, Hong Tao ^a^, Moting Li ^a^, Lingchen Mao ^a^, Zhujun Gu ^a^, Ziyang Ling ^b^, Huancong Shi ^a^*

1. Institute of Environment and Architecture, University of Shanghai for Science and Technology, Shanghai 200093, China

School of Chemistry, The University of Manchester, Oxford Rd, Manchester, UK. M13 9PL

**Support Information**

**Experimental Procedures of the rainfall tests.**

The overall steps of the experiment are provided as follows:

1. A water tank with 20 cm diameter and 20 cm height was placed outdoor, where two samples with standard compressive strength and three samples standard shear strength were put inside. A small beaker and a hyetometer were placed next to the water tank.
2. The hours were recorded during the rainfall until the rain stopped.
3. After getting the samples, the amount of rainwater was measured within a cylinder, and all the experimental instruments were cleaned.
4. The experimental samples of rainwater along with the benchmark were taken from the water tank and small beaker with a syringe filter with a diameter of 0.45 μm.

Data of Fig.2 UCS comparison of standard curing condition and rainfall conditions, 300 kPa was selected as benchmark according to Resource Conservation and Recovery Act (RCRA) of the United State

| Curing time | Standard curing conditon | Error bar | After rainfall test | Error bar |
| --- | --- | --- | --- | --- |
| 3 | 163.26779 | 6 | 178.11032 | 6 |
| 7 | 259.74421 | 8 | 170.68905 | 9 |
| 14 | 489.80337 | 16 | 371.06316 | 13 |
| 28 | 564.016 | 19 | 519.48842 | 18 |

Data of Fig.3 Variation of leachate pH during the semi-dynamic leaching tests

| Leaching time（d） | pH=7 | pH=4 | pH=2 |
| --- | --- | --- | --- |
| 0 | 7 | 4 | 2 |
| 0.5 | 9.02 | 10.88 | 2.49 |
| 1.5 | 9.45 | 11.11 | 2.57 |
| 3.5 | 9.14 | 11.25 | 2.69 |
| 6.5 | 9.1 | 11.46 | 2.77 |
| 10.5 | 8.58 | 11.36 | 3 |
| 15.5 | 8.3 | 11.17 | 2.8 |
| 34.5 | 8.1 | 11.2 | 9.98 |

Data of Fig.5 CFL of target heavy metals (%) under different pH conditions: (a) Cu; (b) Pb; (c) Cd.

| CFL of Cu (%)  Leaching  time（h） | pH=7 | Error bar | pH=4 | Error bar | pH=2 | Error bar |
| --- | --- | --- | --- | --- | --- | --- |
| 12 | 0.00258 | 0.00024 | 0.00379 | 0.00051 | 0.07070 | 0.00928 |
| 36 | 0.00601 | 0.00063 | 0.01355 | 0.00095 | 0.14998 | 0.01897 |
| 84 | 0.00919 | 0.00105 | 0.02888 | 0.00359 | 0.23798 | 0.02432 |
| 156 | 0.01222 | 0.00287 | 0.05229 | 0.00525 | 0.33641 | 0.00383 |
| 252 | 0.01372 | 0.00145 | 0.07787 | 0.00943 | 0.43742 | 0.05067 |
| 372 | 0.01456 | 0.00163 | 0.10274 | 0.01993 | 0.52300 | 0.06382 |
| 828 | 0.01892 | 0.00224 | 0.15912 | 0.02104 | 0.64088 | 0.05674 |

Data of Fig. 5b Pb

| CFL of Pb (%)  Leaching  time（h） | pH=4 | Error bar | pH=2 | Error bar |
| --- | --- | --- | --- | --- |
| 12 |  |  | 0.02488 | 0.00234 |
| 36 | 0.00123 | 0.00013 | 0.10860 | 0.01580 |
| 84 | 0.00519 | 0.00053 | 0.20868 | 0.02382 |
| 156 | 0.01431 | 0.00154 | 0.31798 | 0.04907 |
| 252 | 0.02082 | 0.00255 | 0.41880 | 0.06340 |
| 372 | 0.02636 | 0.00267 | 0.47827 | 0.06269 |
| 828 | 0.03151 | 0.00689 | 0.58543 | 0.04923 |

Data of Fig. 5c Cd

| CFL of Cd (%)  Leaching  time（h） | pH=2 | Error bar |
| --- | --- | --- |
| 12 | 0.06188 | 0.00738 |
| 36 | 0.17964 | 0.01714 |
| 84 | 0.35805 | 0.05306 |
| 156 | 0.63310 | 0.08543 |
| 252 | 1.02292 | 0.19521 |
| 372 | 1.42600 | 0.28536 |
| 828 | 1.66690 | 0.35425 |
